# Malthusian Parameters as Estimators of the Fitness of Microbes: A Cautionary Tale about the Low Side of High Throughput

**DOI:** 10.1371/journal.pone.0126915

**Published:** 2015-06-26

**Authors:** Jeniffer Concepción-Acevedo, Howard N. Weiss, Waqas Nasir Chaudhry, Bruce R. Levin

**Affiliations:** 1 Department of Biology, Emory University, Atlanta, Georgia, United States of America; 2 Department of Mathematics, Georgia Institute of Technology, Atlanta, Georgia, United States of America; 3 National University of Sciences and Technology, Islamabad, Pakistan; Leiden University, NETHERLANDS

## Abstract

The maximum exponential growth rate, the Malthusian parameter (MP), is commonly used as a measure of fitness in experimental studies of adaptive evolution and of the effects of antibiotic resistance and other genes on the fitness of planktonic microbes. Thanks to automated, multi-well optical density plate readers and computers, with little hands-on effort investigators can readily obtain hundreds of estimates of MPs in less than a day. Here we compare estimates of the relative fitness of antibiotic susceptible and resistant strains of *E*. *coli*, *Pseudomonas aeruginosa* and *Staphylococcus aureus* based on MP data obtained with automated multi-well plate readers with the results from pairwise competition experiments. This leads us to question the reliability of estimates of MP obtained with these high throughput devices and the utility of these estimates of the maximum growth rates to detect fitness differences.

## Introduction

With the advent of synthetic biology and ever-improving tools of molecular biology, experimental evolution studies with bacteria and yeast have been, and will continue to be, an increasingly important and powerful way to draw inferences about the genetic and molecular basis of adaptive evolution. Central to these inferences are estimates of the fitness of the constructed and/or evolved microbes relative to their ancestors or other base strains.

Commonly, fitness is estimated from the changes in the frequencies of strains grown together, often referred to as pairwise competition experiments [1–8]. Unfortunately estimating relative fitness in this way is a labor-intensive, time- and supply- consuming endeavor. Moreover, if colony forming unit (CFU) data are used to estimate the densities and relative frequencies of the competing populations, even with considerable replication, it is not possible to detect relative fitness differences of much less than 1%. Using bacteria that express fluorescent proteins and a flow cytometer, quite small differences in relative fitness can be detected with pairwise competition experiments [9,10]. This method for estimating fitness is particularly appealing for in vitro experimental evolution studies with well-characterized laboratory strains of microbes [11], but would be difficult to apply for surveys of fitness costs for many different species and strains.

One alternative to pairwise competition experiments to measure fitness differences among strains of microbes has been to estimate the maximum exponential growth rates, the Malthusian Parameter (MP) of these strains [12–20]. In the past, estimating MPs from sequential samples of replicating cultures of microbes with CFU or optical density was an intense and tedious enterprise. This was particularly so if the experiments were done with sufficient replication to detect small strain differences in the magnitude of this parameter. Thanks to the advent and wide availability of microtiter plate readers with incubated chambers that shake the culture to aerate and re-suspend the cells, investigators measuring growth rates have been freed of this tedium. Since these devices can be programmed to automatically record the optical densities at predefined intervals, one can simultaneously estimate MPs with a great deal of replication for large numbers of strains or species of planktonic microbes, high throughput even.

In this report, we consider the reliability and reproducibility of estimates of MP obtained with automated microtiter plate readers and evaluate the utility of estimates of this parameter for drawing inferences about the relative fitness of bacteria. Using antibiotic resistant mutants or transconjugants of *Escherichia coli*, *Pseudomonas aeruginosa*, and *Staphylococcus aureus*, we explore (i) the absolute and relative estimates of MPs using two different microtiter plate readers, and (ii) the extent to which MP estimates of relative fitness are able to detect differences in fitness among strains observed in pairwise competition experiments. The results of our study question the reliability of microtiter plate reader estimates of MPs and their value for drawing inferences about the relative fitness of bacteria.

## Materials and Methods

### Bacteria

1. *E*. *coli* B (Designated **BAM**)—*tsix rpsL*, *ara R-M-* (obtained in 1969 from S. Lederberg in 1969).
  - **BAP—**a spontaneous Ara+ mutant derived from *E*. *coli* B/6
  - **BAM-Nal**- a spontaneous nalidixic acid (gyrA) resistant mutant of B/6Ara-
  - **BAM-JCA**—B/6Ara-(pJCA)—A transconjugant of B/6Ara- bearing a conjugative (Tet Amp) plasmid obtained from the *E*. *coli* flora of one of the authors.
2. Ps*eudomonas aeruginosa* PA14 obtained from (J. Goldberg), designated **PA**
  - **PA-CIP** a spontaneous ciprofloxacin resistant mutant of PA14
3. *Staphylococcus aureus* Newman (obtained from William Shafer), designated **SA**
  - **SA-CIP—**a spontaneous ciprofloxacin resistant mutant of SA
  - **SA-FUS—**a spontaneous fusidic acid resistance mutant of SA

### Media

1. Liquid-
  - Lysogeny Broth (LB) (Difco Ref. #244620) for the *E*. *coli* and *P*. *aeruginosa* strains
  - Cation-adjusted Muller Hinton Broth (MHII) (BBL Ref# 212322) for the *S*. *aureus* strains
  - Glucose (500μg/ml)—limited Davis Minimal Medium [21] supplemented with100μg/ml MgSO and 2μg ml Thymine—for the *E*. *coli* and Pseudomonas strains.
2. Solid media
  - LB-agar, LB with16 gm/L Bacto agar
  - LBCIP—agar, LB with Ciprofloxacin (Sigma) at 1μg per ml for the competition experiments with *S*. *aureus* and 0.3μg/ml for the experiments with *P*. *aeruginosa* and 16 gm/L Bacto agar
  - LBFUS-agar—LB with 2.5 μg/ml Fuscidic Acid (Sigma) and 16 gm/L Bacto agar
  - TA- Tetrazolium Arabinose-agar [2]- indicator media for sampling the *E*. *coli* competition experiments (Ara+ pink, Ara- bright red).

### Preparing the cultures for the growth experiments

Unless otherwise noted, starting from single colonies the bacteria were grown overnight for 20–24 hours in the media used for the growth rate experiments. Cultures were then serially diluted in saline with the final dilution in the medium used for the growth experiment. Using automatic multi-dispensing pipettes (Ranin LTS), the cell suspensions were distributed into the wells of the microtiter plates for a final density of ~10^5^ ml. At these low densities the optical densities (ODs) of the cultures are the same as those in the cell-free wells with that media. Immediately after preparation the plates were placed in the microtiter plate readers and optical densities estimated five minutes.

### The Microtiter plate readers and growth conditions

1. Bio-Tek ELx808 –Flat-bottom 96 well plates with low-evaporation lids (Costar) were, unless otherwise noted, inoculated with 300 μl of cell suspension. The cultures were shaken for 10 seconds at the highest possible level (3) before each sample. Optical densities were estimated at 630 nm at 37°C for 20–24 hours.
2. Bioscreen C—One hundred wells Bioscreen plates were, unless otherwise noted, inoculated with 300μl of the cell suspensions. The optical density for each well was measured at 600 nm for 20–24 hours at 37°C. Unless otherwise noted, save for when the ODs were estimated, the cultures were shaken continuously.

With the BioTek plate reader, there was no setting for continuous shaking and the BioTek and Bioscreen filters that determined the wavelength were different, respectively 630nm and 600nm. To explore the effects of intermittent rather than continuous shaking and that of wavelength we estimated the MP of all wild type bacteria in minimal medium and broth with intermittent shaking and at wavelength set at 540. The results of this experiment are presented in the S4 Fig.

### Pairwise competition experiments

Overnight cultures derived from single colonies of the two competing clones were mixed, 20μl of each, into 4 ml of medium in 12 well macrotiter plates (Costar). These mixed cultures were then dilutes 1:100 in fresh medium and grown second time and the densities and relative frequencies of the competing clones estimated, time 0. The cultures were then transferred to three wells (see the diagram in S1 Fig). The purpose of growing the cells together before starting the competition experiment is to minimize the effects of starting conditions. The bacteria in the three wells derived from the mixture were separately transferred and estimates of the densities and relative frequencies of the competing populations made at each transfer for 4 or 5 transfers.

### Estimating the Malthusian Parameter

#### Normalization

Since we are interested in estimating the rate of change in the density of bacteria, dN/dt, and are only using the optical density of the culture (light scattering) as surrogate for N, we have to assume some relationship between N and OD. As is traditionally the case, we assume that when the bacteria are growing near the maximum rate, this relationship is linear, so that at any time, t, the optical density of the culture:

$$OD(t)=k\;X\;N(t)+OD_{M}$$

or

$$N(t)=\frac{1}{k}\left[OD(t)-OD_{M}\right]$$

where k is constant and OD_M_ is the optical density of the media. Thus the first step in estimating the Malthusian parameter is to normalize the ODs, by subtracting the OD_M_, which as noted above is the time 0 estimate of the OD for that well.

#### Calculations

Different methods have been employed to estimate the Malthusian parameter (MP) from optical density data, e.g. [17,22]. Here we use the Growth Rates regression-method program developed by Barry Hall and colleagues; see [23] for details about this method. We have made two modifications of their suggested protocol. First, for normalization we use the time 0 estimates of the optical densities for each well, rather than their suggested average for cell-free wells in that plate. As noted earlier, using initial densities of bacteria on the order of 10^5^ or lower, the light scattering off these cultures is below the detection limit of both instruments and the OD of the cultures are not statically significantly different than that in cell-free media OD_M_. Second, we initiate our calculations of the MP at a normalized OD of 0.02±0.002. The reason for the latter can be seen from the results of the Calibration experiments in the following section.

### Statistical Analyses

We use one-factor and two-factor analyses of variance to test for differences in the estimated MPs between cultures and microtiter plate readers. For our consideration of the microtiter plate reader (machine)–associated differences in relative growth rates of wild type and resistant mutants, we use the bootstrap method described in the S1 Supporting Information.

## Results

### Calibration Experiments

#### Culture Volume

**Fig 1:**
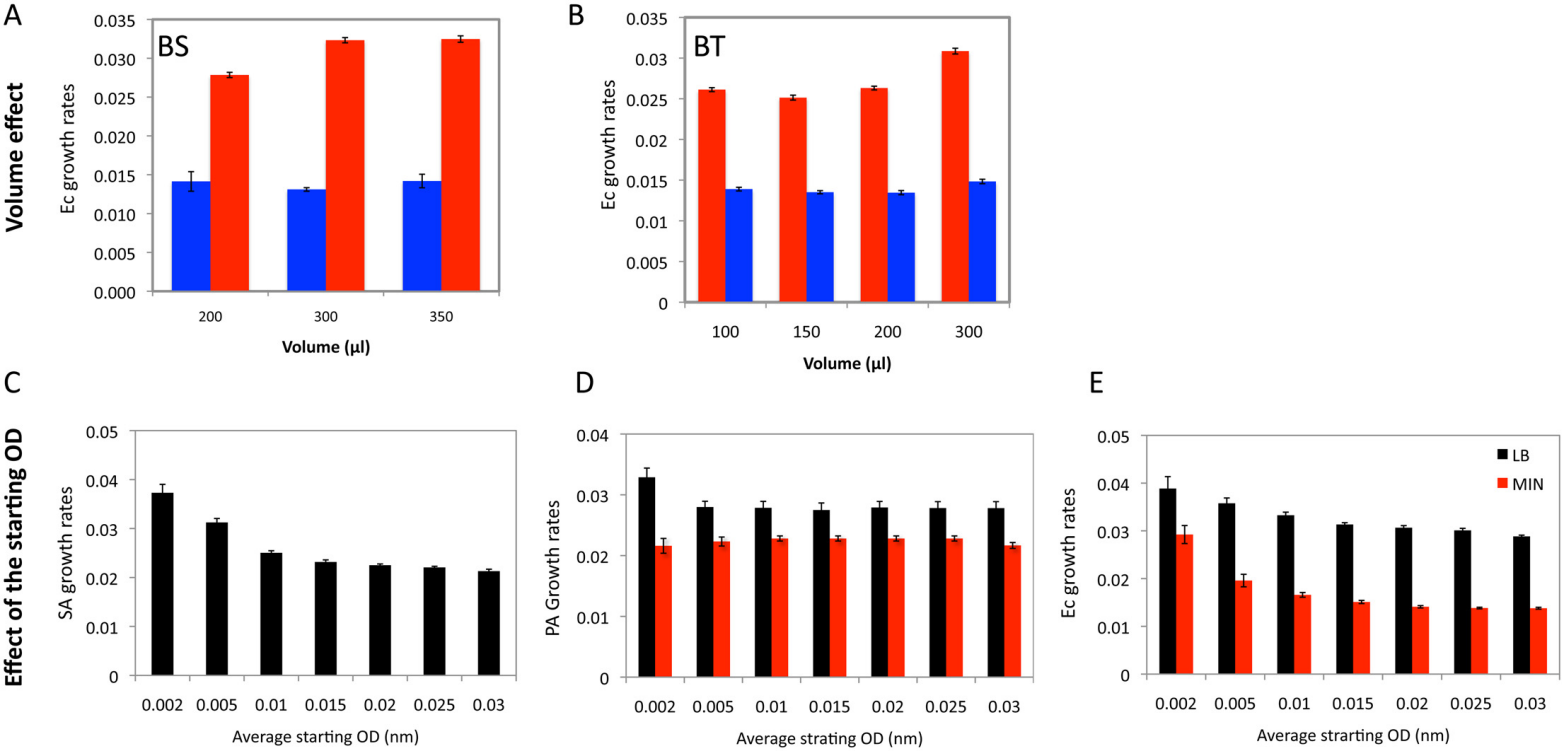
Effects of well volume and initial OD on the estimated MP. (A, B) Effects of volume on estimated MP, respectively Bioscreen (n = 10) and BioTek (n = 12). *E*. *coli* WT in glucose-limited (blue) and LB (Red). Mean and standard error: Bioscreen LB F(2,27) = 820.5, p< 0.001, minimal, F (2,27) = 66.6, p<0.0001. Bio-Tek LB F(3, 28) = 78.8, p<0.001, Minimal, F(2,27) = 7.17, p~0.005). (C, D and E) effect of starting OD on the estimated MP, (C) Wild type *S*. *aureus* in MHII. (D and E), respectively for wild type *E*. *coli* and *Pseudomonas aeruginosa* in LB broth (black bar) and minimal media (red bar).

To explore the effects of the volume of bacterial suspension in the wells of the microtiter plates on the estimated Malthusian Parameter using the Bioscreen and BioTek data and the Growthrates software, we calculated the MP for *E*. *coli* in glucose-limited minimal medium and LB with different volumes of the cells suspensions in the wells. As suggested in [23] and can be seen in Fig 1A and 1B and S1 Table for the statistical analysis, the volume of media in the wells can contribute to the variation in the estimates of the Malthusian parameter. Based on these results, in the experiments that follow, we used 300μl in each well for both the BioscreenC and the BioTek.

#### Initial OD

On first consideration, it would seem that if the density of bacteria were below that detected by the plate readers, their populations would be growing at the maximum rate when they reach this OD threshold. Consequently, it should be possible to use the normalization OD (when OD = 0) as the initial optical density for the reliable estimates of the MP of exponentially growing populations of bacteria. The results of our experiments indicate that this is not the case and that the starting OD required to obtain reliable estimates of MPs with a Microtiter plate reader and the Growth Rates software varies among species of bacteria (Fig 1C–1E.

Using the Bioscreen data and the Growth Rates program we estimated the maximum growth rate for different initial ODs in glucose (500μg/ml) minimal medium and Lysogeny Broth (LB) for *E*. *coli* and *P*. *aeruginosa*, and in MHII broth for *S*. *aureus*. The results of these experiments are presented in Fig 1C–1E.

Estimates of the MP obtained with the lowest normalized starting OD (0.002) are, save for *P*. *aeruginosa* in minimal medium, greater than obtained with somewhat higher starting ODs. For *P*. *aeruginosa* in broth as well as minimal medium, there was no statistically significant difference in the estimates of MP obtained with a starting OD of 0.005 and a starting OD of 0.03. This leveling off in the estimated MP did not occur for the *Staphylococcus aureus* or *E*. *coli* strains until the starting OD was 0.015. For this reason, in calculating the MPs in this study, we use the normalized OD value of 0.02 ±0.002 for the starting optical density for our calculation with Growthrates software. Albeit not central to this consideration, it is of some interest to note that the Malthusian parameter for *P*. *aeruginosa* PA14 in minimal medium (Fig 1D, red bars) is about 80% that in LB (Fig 1D, black bars), whilst for *E*. *coli* B/6 the rate of growth in minimal is about 46% that in LB (Fig 1E).

#### Age of the inoculum, replication, well location, shaking and wave length

**Fig 2:**
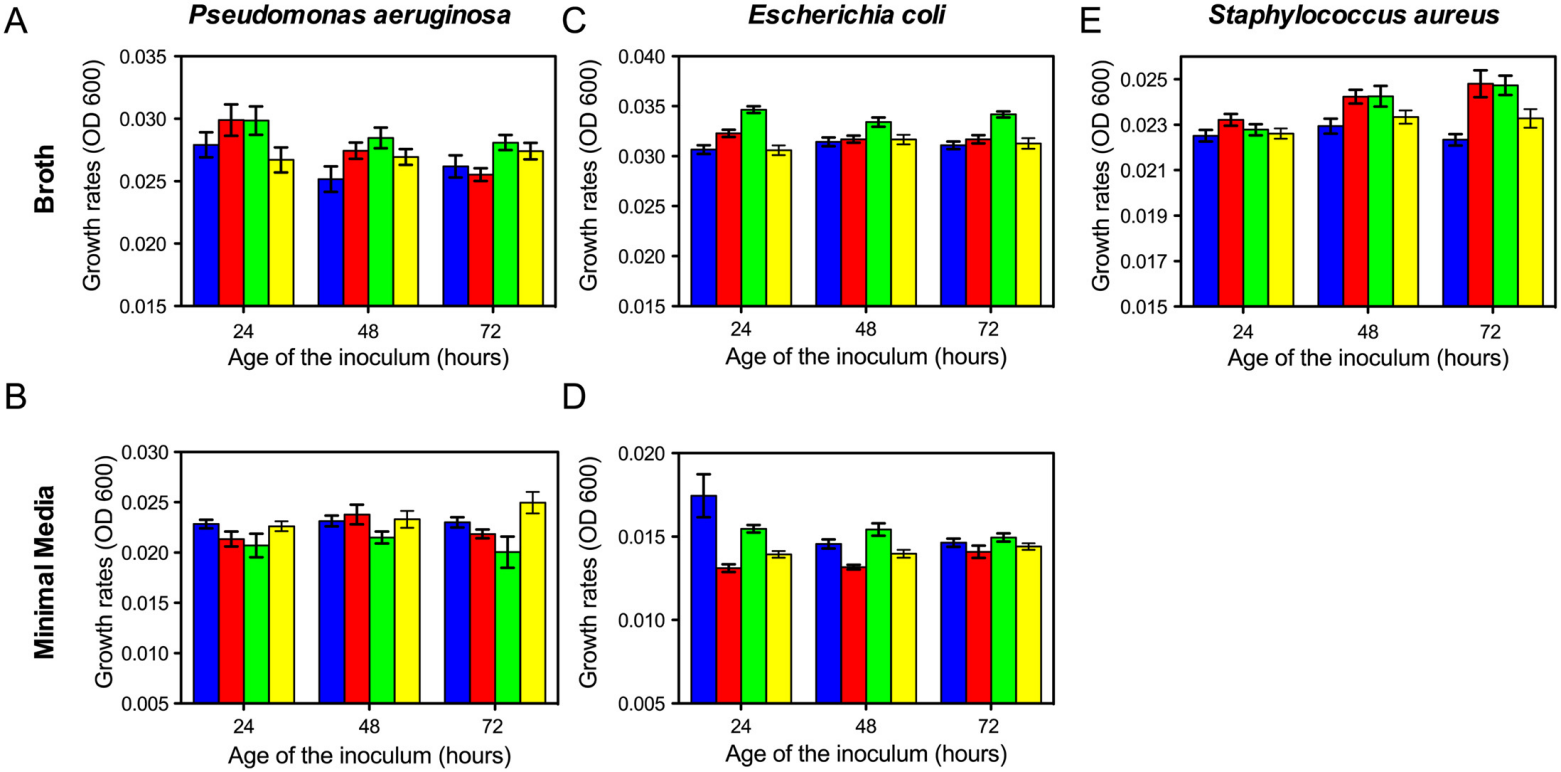
Variation in estimated MP attributable to the age of the culture and replica. Each color represents a different replica and the values represent the average OD from 10 different wells. (A and B) respectively, *Pseudomonas aeruginosa* WT in LB and glucose limited minimal medium. (C and D) respectively *E*. *coli* WT in LB and glucose-limited minimal medium. (E) *S*. *aureus* Newman, WT, in MHII. For statistics refer to S2 Table.

In their protocol, Hall and colleagues [23] recommend careful control of the preparation, and in particular the age of the inoculum introduced into the microtiter wells. They suggest there will be variation in estimated MPs between runs and within microtiter plates. To explore the magnitude of this variation, and thereby the robustness and reliability of microtiter plate reader estimates of the MP, we estimated this parameter with suspensions of wild type cells of each species initiated from cultures maintained for 24, 48, and 72 hours at 37°C, in their respective media. We repeated this experiment four times to determine the extent to which estimates of MP vary between runs. We also examined the extent of variation within plates by inoculating 50 wells with the same cell mixture (*E*. *coli* B/6 in LB). We restricted this consideration to the Bioscreen data. The results of these experiments are presented in Fig 2. Finally, to determine the effects of the wave-length of the plate reader and the extent of shaking, using the Bioscreen we estimated the MPs for wild type strains of all three species in broth and minimal medium at a wavelength of 540nm and with shaking for 10 seconds before reading the ODs, rather than continuously between readings.

For all three species, there is statistically significant variation in the estimated MPs between runs in broth (Fig 2A–2E) and for *E*. *coli* and *P*. *aeruginosa* in glucose limited minimal medium as well (Fig 2B and 2D). Significant age of inoculum effects obtained for *E*. *coli* in LB and *S*. *aureus* in MHII. The only statistically significant interaction between the age of the inoculum and replica is for *E*. *coli* in minimal medium (p~0.0005) despite no significant age of culture effect (p~0.078) (Fig 2D).

For all three species in broth (MHII or LB) and minimal medium for *E*. *coli* and *P*. *aeruginosa*, there is a statistically significant difference in the estimates of MP between runs. Statistically significant ages of inoculum effects were obtained for Pseudomonas in LB, but not in minimal medium, and for *S*. *aureus* in MHII.

To determine the relationship between the age of the inoculum and MP for these Bioscreen data, we performed a regression analysis for these two cultures with statistically significant age effects using the data from all four replicas (S2 Fig). For the *S*. *aureus* there is a statically significant positive correlation with MP and the age of the culture (r = 0.031) with a linear regression coefficient of 2x10^-5^ (9x10^-6^ to 3.3x10^-5^, 95% confidence interval). For the *P*. *aeruginosa* in LB, the correlation coefficient is r = -0.25 and the linear regression coefficient -4x10^-5^ (-6 x10^-5^ to 1x10^-5^, 95% confidence interval). No statistically significant differences in estimates of MP were detected between columns and plates when MPs were calculated from the same run (S3 Fig).

For *E*. *coli* in and *S*. *aureus*, the estimated MP obtained with the Bioscreen set at 540nm with continuously shaking and at 600nm with the cultures shaken for 10 seconds before reading (Intermittent) were not significantly different than that obtained with the standard continuous shaking 600nm protocol (S4 Fig). For *P*. *aeruginosa*, however, the estimated MP obtained with intermittent shaking were significantly greater than that obtained with continuous shaking in the Bioscreen. Moreover, the ratio of the standard error to the mean for the Pseudomonas estimates of MP is substantially greater than that for *E*. *coli* and *S*. *aureus*.

### II. Instrument variation and the relationship between Malthusian parameter and pairwise competition estimates of fitness

There are a variety of incubated microtiter plate readers that can be used to automatically follow the dynamics and estimate the parameters of bacterial growth from OD data. As noted in the Methods section, we did these growth rate experiments with two of these machines, a Bioscreen and a BioTek. In this section, we consider the magnitude of the absolute and relative differences between these incubated plate readers in the estimated Malthusian parameter and the extent to which this parameter predicts the competitive performance of these bacteria. We used wild type strains of *E*. *coli* B/6, *S*. *aureus* Newman, and *P*. *aeruginosa* and the mutants and transconjugants of these and the media described in the methods section.

**Fig 3:**
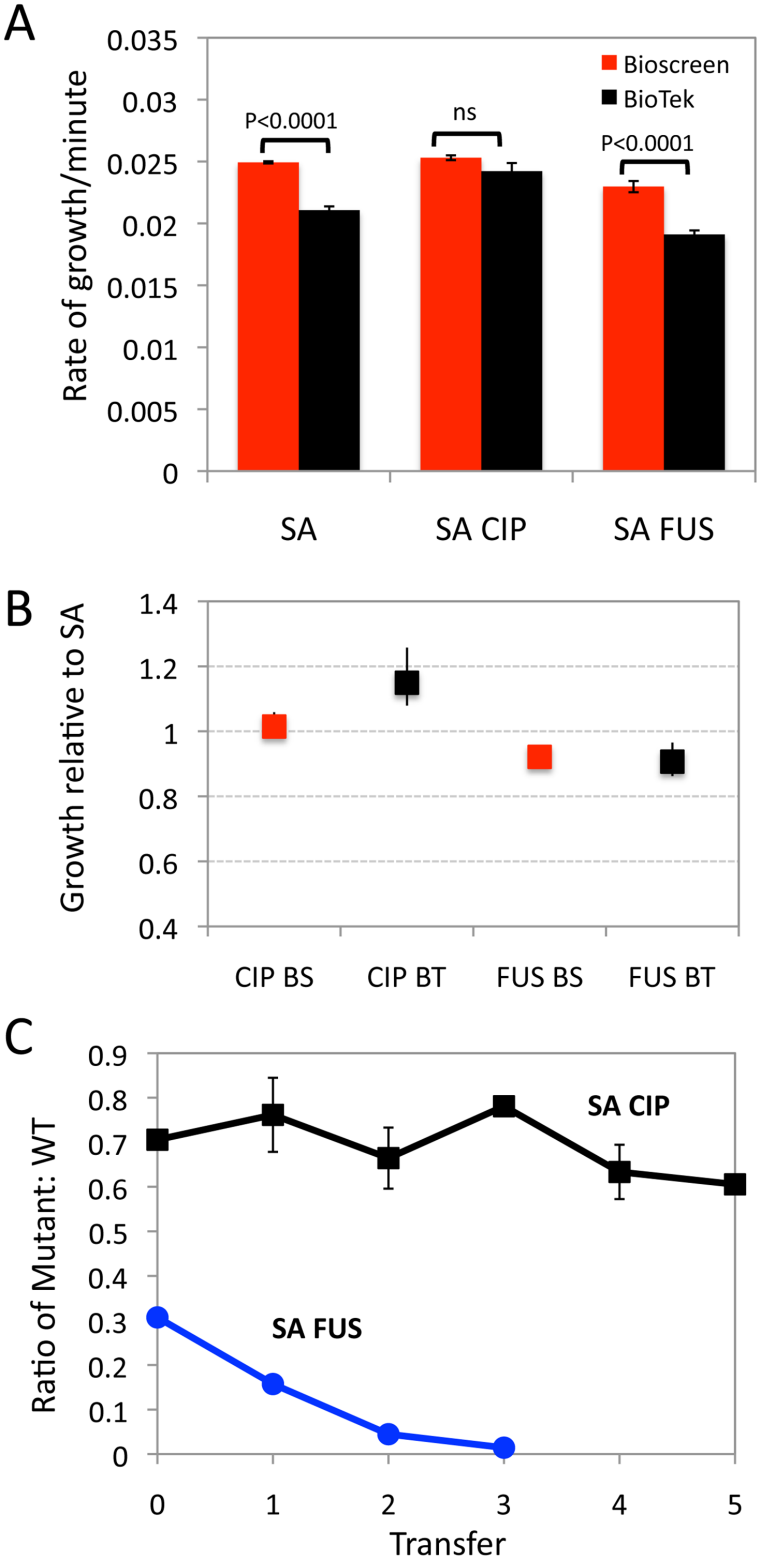
**Malthusian parameter and pairwise competition estimates of fitness for *S*. *aureus* ciprofloxacin and fusidic acid susceptible and resistant mutants** (A) Growth rates estimates from three *S*. *aureus* strains using a Bioscreen (BS, red bar) and BioTek (BT, black bar) plate readers. SA is an antibiotic sensitive strain, SA-CIP is a ciprofloxacin resistant strain and SA-FUS is a fusidic acid resistant strain. (B) Malthusian parameters relative to wild type (SA), (Bioscreen red BioTek black) and Bootstrap-calculated confidence intervals. (C) Changes in the ratio of the antibiotic resistant mutants and SA WT in pairwise competition SA-CIP (black) and SA-FUS (blue), mean and standard deviation of the ratios from three independent competition experiments.

*S*. *aureus*: For two of the three strains examined (SA and SA-FUS) the absolute value of the MP estimated in the Bioscreen is significantly greater than the corresponding estimate in the BioTek (Fig 3A). Considering the bootstrap 99% confidence intervals (Fig 3B) there are no statistically significant machine differences in the relative to WT MPs of the SA-FUS mutant. This was not the case for the SA-CIP mutant, where the relative to WT estimate of the MP in the BioTek is significantly greater than the corresponding estimate obtained with the Bioscreen. The estimates of the MP obtained with both machines predict a fitness cost for the fuscidic acid resistance mutant (SA-FUS). Our pairwise competition results support this prediction (Fig 3C, blue line). For the SA-CIP, the pairwise competition experiments predict little or no fitness cost for the ciprofloxacin resistance mutant (Fig 3C, black line). This is consistent with what is anticipated from the relative MP estimates obtained in the BioScreen (Fig 3B), whilst the BioTek relative growth rate difference suggests a fitness advantage for the Ciprofloxicin resistant mutant.

**Fig 4:**
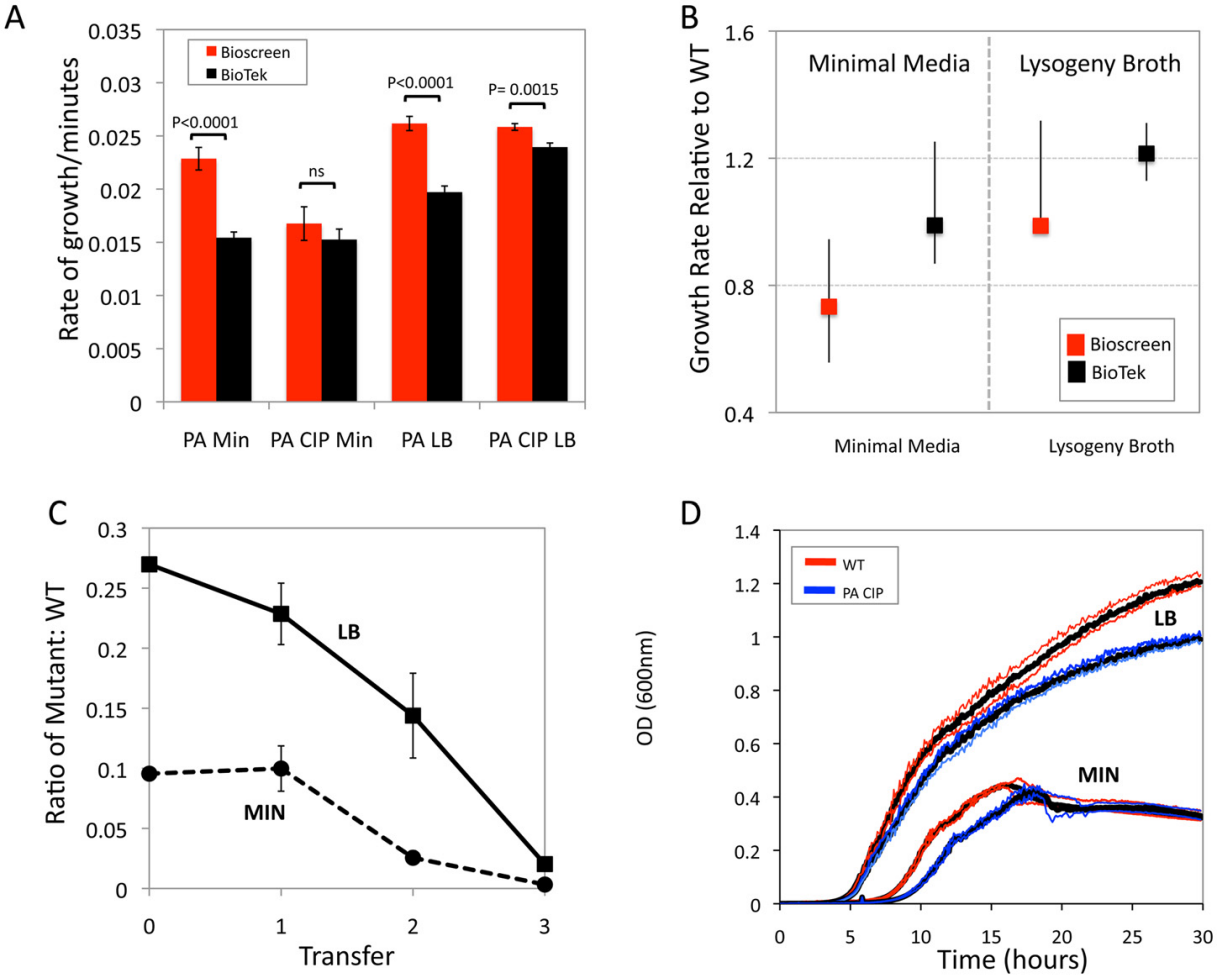
Malthusian parameter and pairwise competition estimates of fitness for *Pseudomonas aeruginosa* ciprofloxacin susceptible and resistant mutants in LB and glucose-limited minimal medium. (A) Estimates of the absolute MP of WT and PA-CIP obtained with a Bioscreen (red bars) and BioTek (black bar) plate readers. (B) MP of PA-CIP of relative to wild type *P*. *aeruginosa* in glucose-limited minimal medium and LB, Bioscreen red and BioTek black. (C) Pairwise competition, ratio of PA-CIP strain relative to wild type in LB and minimal medium, mean and standard deviation of the ratios from three independent competition experiments. (D) Longer term changes in OD of CIP resistant and sensitive strains in minimal medium and LB, Bioscreen data.

*Pseudomonas aeruginosa*: In minimal media (Min) the absolute estimate of MP for PA obtained in the Bioscreen is significantly greater than that in the BioTek but not for PA-CIP. For both strains, the MP estimated in LB is significantly greater in the Bioscreen than the BioTek (Fig 4A). Based on the 99% Bootstrap estimates of the confidence intervals, in neither minimal medium nor broth is there a significant difference in MP for the CIP mutants relative to WT (Fig 4B). Whilst for the Bioscreen the 99% Bootstrap estimate of the MP for PA-CIP relative to WT predicts a fitness cost, an MP < 1.0, with this resistant mutant in minimal medium. No fitness difference is anticipated in minimal medium for the BioTek estimate of MP. These estimates of MP predict no fitness cost of the PA-CIP mutation in LB. However, the pairwise competition experiments demonstrate a fitness cost for ciprofloxacin resistance in both minimal medium and LB. At least part of the reason for the failure of the estimates of MP in LB to detect the lower relative fitness of the CIP-R mutant in pairwise competition with WT can be seen in Fig 4C. Although there is no initial difference, as time proceeds, the rate of growth (as measured by OD changes using the Bioscreen) of the CIP mutant declines at greater rate than the wild type (Fig 4D).

**Fig 5:**
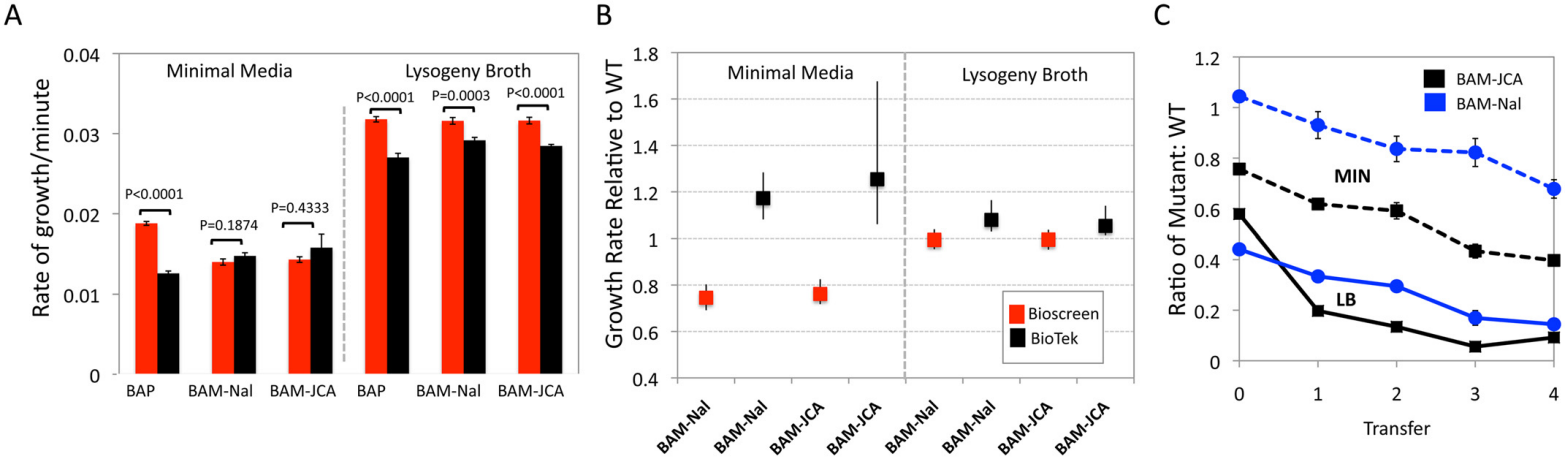
Malthusian parameter and pairwise competition estimates of fitness for *E*. *coli* antibiotic sensitive and resistant mutants and transconjugants in LB and glucose-limited minimal medium. (A) Estimates of the MP in minimal medium (left panel) and broth (right panel), Bioscreen (BS, red bar) and BioTek (BT, black bar). (B) Relative to WT growth rates of BAM-Nal and BAM-JCA in minimal and broth. (C) Ratio of antibiotic sensitive (WT) and resistant mutants in pairwise competition; BAM-Nal and WT (blue) and BAM-JCA and WT (black). Mean and standard error for 3 independent cultures from the same initial mixture.

*E*. *coli*: The MP estimated from the BioTek data is significantly lower than that obtained with the Bioscreen in both Minimal medium and LB (Fig 5A). There is no machine-associated difference in the ratio of the MP of the Nal-R (BAM-NAL) mutants and pJCA transconjugants (BAM-JCA) relative to WT (BAM) in LB, or an anticipated fitness cost of these mutants and transconjugants in LB (Fig 5A). On the other hand, in accord with the bootstrap estimates of the 99% confidence intervals, there is a highly significant difference in this ratio for the estimates of the MPs of these strains in minimal medium. Most critically, whilst the BioTek data predict no fitness cost for the resistant strains in minimal media, a substantial fitness cost of these resistant mutants and transconjugants is anticipated in minimal medium from the Bioscreen date but not in LB (Fig 5B). The pairwise competition experiments between these resistant clones and WT reveal a fitness cost for the BAM-Nal mutant and BAM-JCA transconjugants in both minimal media and broth (Fig 5C).

## Discussion

Multi-well plate readers which automatically record OD measurement, incubate, and shake cultures, along with the Growthrate software developed by Barry Hall and colleagues [23], offer an easy way to estimate the maximum exponential growth rates of planktonic populations of microbes, the Malthusian parameter (MP). With little hands on effort and lots of replication these estimates can be obtained for large numbers of clones and species of planktonic microbes, "high throughput".

We believe that the technical recommendations made in this report further facilitate this automated method of estimating this parameter. By inoculating the wells with densities of bacteria below the plate reader detection threshold, the ODs at time 0 for each well can be used to normalize the data to control for the contribution of the media to the optical density. Thus media-only cell-free blanks are not required and the OD_M_ of the media used for normalization is that for the well from whence the growth data comes. By initiating the calculations with normalized ODs of 0.02 ± 0.002, estimates of MPs are constant over a reasonable range.

### Other growth parameters

Although they can contribute to the fitness of bacteria, in the body of this paper we have limited this consideration to the Malthusian Parameter and have not considered the contribution of the Lag or Maximum OD to fitness. These parameters can be estimated with automated plate readers and the Growthrates software. To estimate the Lag, it is necessary to do the regression with normalized time t = 0 OD as the initial point. While for minimal medium the maximum OD peaks and levels off, for broth it continues to increase (see Fig 4D) over a 24 hour period. As can be seen in S5 Fig, where we used the data from the Bioscreen age of inoculum experiments depicted in Fig 2, as measured by the relatively small standard error, the estimates of the lag and maximum OD obtained this way are reliable.

### Absolute and relative growth rates, replication, position and age of inoculum effects

It is not surprising that the estimates of the absolute value of MP obtained with Bioscreen and the BioTeK microtiter plate readers differ. While these machines were both set for 37°C, there may well be machine differences in temperature control, which could affect growth rate. It is also not unexpected that for most of the strains of bacteria examined, the estimated MP obtained with the Bioscreen exceeds that from the BioTek. These are aerobic bacteria and because the cultures are shaking continuously in the former plate reader, they may well be better aerated than they are in the BioTek, as well as better-suspended and less likely to form biofilms. Indeed, we consider biofilm formation as a likely explanation for why in the Bioscreen intermittent rather yields higher estimates of MP than continuous shaking. Also consistent with this interpretation is the substantially greater ratio of the standard error to the mean for Pseudomonas.

Arguably, machine differences in the estimates of MP would not be a problem for fitness estimation, if different microtiter plate readers provided the same relative estimates of the MPs for the strains under consideration. This was not the case. Not only did these microtiter plate readers provide statistically significantly different estimates of the absolute MP for both the *E*. *coli* and *S*. *aureus*, there were significant machine differences in the ratio of the estimated growth rates for wild type and antibiotic resistant strains. Stated another way, inferences about the magnitude and even the direction of fitness differences based on MP estimates made with microtiter plate readers can depend on the plate reader employed in the study and the manner and extent to which they aerated cultures, re-suspend the cells, and possibly other factors.

It is critical to point out that this study is restricted to estimates MPs from optical density data obtained with automated microtiter plate readers. There are other methods to estimate this parameter. We have had experience with estimating MPs from colony count data and from optical densities estimated “by hand” from growing cultures. The variance in estimated MPs one obtains with this time-consuming and tedious approach considerably exceeds obtained with automated plate readers. MPs can also be estimated with fluorophores, luminescent reporters, particle counters and flow cytometers. We have no experience with these methods for estimating MPs and are unaware of studies similar to this one to evaluate their reliability and precision for estimating this parameter for multiple species or strains or evaluating the utility MP estimated in these ways for detecting fitness differences observed in pairwise competition experiments. If, however, fitness differences are due to factors other than maximum growth rate, then no matter what method is employed to estimate MP, these differences will not be detected by concentrating on this parameter alone.

#### Malthusian parameters and pairwise competition experiments to detect fitness differences

The observation that the transconjugants and most of the antibiotic resistant mutants used in this study had a fitness cost is not unexpected. These were newly generated transconjugants and mutants and there was not sufficient time for selection to ameliorate these costs [7,24,25]. It is also not surprising that differences in the relative performance of the wild type strains of these species and their resistant mutants that can be detected in pairwise competition experiments are not anticipated from differences in estimated MPs of these strains. The maximum growth rate is only one element of the competitive performance of bacteria. No matter what method is employed to estimate MPs and how precise those estimates are, this parameter may not be sufficient to detect inherited differences in fitness that could be revealed in pairwise competition experiments. Differences in lag, the approach to and levels of stationary phase, and rates of mortality at stationary phase all contribute to the relative performance of bacteria in pairwise competition experiments. Moreover, there can also be synergistic and antagonistic interactions between the competing strains that could also contribute to their performance in mixed culture that would not be detected in the monocultures from whence we are estimating MPs.

### Mechanistic questions not addressed and why

The motivation for this study and its goal is to critically evaluate the reliability of estimates of the maximum exponential growth rates of bacteria, the Malthusian parameter, MP, obtained with microtiter plate readers, and the utility of these estimates for detecting fitness differences among strains of bacteria. We consider this investigation as a technical contribution, as a service to the community of scientists using microtiter plate readers to estimate growth rates and particularly colleagues using these data to estimate relative fitness. This study was not intended to increase our understanding of the dynamics of bacterial growth and fitness or how antibiotic resistance encoding genes and plasmids affect the fitness of bacteria. Be that as it may, in the course of this investigation we noted a number of intriguing mechanistic questions and have no doubt the readers will see these and more. While we believe that addressing these questions is “beyond the scope” of this technical contribution, we hope the readers will find some of them sufficiently interesting to pursue. Included among the mechanistic questions we see are:

1. Why does initiating the regression analysis with normalized ODs of less than 0.015 for *E*. *coli* and *S*. *aureus* lead to substantially higher estimates of the MP than those initiated at an normalized OD greater than 0.015?
2. Why can one get reliable sustained estimates of the MP for *P*. *aeruginosa* with lower initial ODs than one can for *E*. *coli* or *S*. *aureus*?
3. Why there is an effect in the amount of time a culture has been in stationary phase on its estimated MP once it starts growing?
4. Why do the relative estimates of the MPs of the wild type and otherwise isogenic antibiotic resistant mutants and transconjugants differ between microtiter plate readers? Could it be that these differences reflect environmental factors that contribute not only to the fitness effects of antibiotic resistance but fitness in general?
5. To what extent can the observed difference in competitive performance of the antibiotic susceptible and resistant strains be attributed to differences in the MP?
6. Why do the antibiotic resistance mutations and/or the carriage of resistance-encoding plasmids engender a cost to the fitness of the bacteria?
7. Why is the substantial fitness cost observed for the ciprofloxacin resistant mutant of *P*. *aeruginosa* not manifest for the ciprofloxacin resistant mutant of *S*. *aureus*?
8. Why is there more variation in the estimates of MP for *Pseudomonas aeruginosa* than *E*. *coli* or *Staphylococcus aureus* and why are the estimated MPs for Pseudomonas greater with intermittent shaking than with continuous shaking?

## Conclusions and Recommendation

A failure to detect fitness differences by any experimental method is, of course, just a negative result. There may well be fitness differences in other culture media, at different temperatures, pH or other environmental conditions. For pairwise competition experiments, fitness differences may be observed at different densities or with the competing populations at different initial frequencies [26]. There is also the possibility that estimates of fitness obtained for microbes grown in physically structured habitats (an inconvenient but all too common reality) may not be reflected in experiments performed with planktonic cells [27,28]. On the other hand, if statistically significant fitness differences are observed for microbes in the laboratory culture, we can say with assurance that there are conditions under which those strains will differ in fitness. Whether those conditions exist in the world beyond our flasks, chemostats and Petri dishes is another matter.

For the two reasons considered above, (a) machine variation in the relative estimates of MPs and, (b) the failure to detect fitness differences that are readily observed in pairwise competition experiments, we do not endorse the use estimates of the MP obtained with microtitre plate readers as a unique method to detect fitness differences, much less estimate the magnitude of those differences.

To be sure, the ease of estimating MPs with automated microtiter plate readers is appealing. Estimates of the maximum growth rates of planktonic microbes in single clone culture obtained with these high throughput devices may be useful for initial screening for fitness differences among strains of bacteria. We would anticipate that substantial difference in MPs estimated with microtiter plate readers would be reflected as fitness difference in pairwise competition experiments. In our opinion, however, pairwise competition experiments are the most effective method for detecting fitness differences in bacteria, estimating their magnitude, and determining the ecological, microbiological, and genetic and molecular reasons for these differences.

## Supporting Information

S1 Supporting Information Malthusian Parameters as Estimators of the Fitness of Microbes: A Cautionary Tale about the Low Side of High Throughput. Method used for the pairwise competition experiments,Calibration experiments not included in the body of this article,The bootstrap method to estimate the confidence intervals in relative maximum exponential growth rates and the Mathematica code for computing these intervals.
(DOCX)

S2 Supporting Information Data Set. Raw data files for this study.
(ZIP)

S1 Fig Diagram of the pairwise competition experiments employed in this study. Overnight stationary phase cultures of the competing strains were mixed on day two, inoculated in media for a ratio 1:100 and grown together for 24 hours. Samples were taken to estimate the densities and relative frequencies of the competing strains, Time 0, and inoculated into 3 wells, 1:100 with media. This process of sampling and dilution in fresh media was repeated for 4 transfers.
(TIF)

S2 Fig Effect of the age of the culture. Effect of the age of the culture on the estimated MPs for *Staphylococcus aureus* (A) and *Pseudomonas aeruginosa* (B). Estimated MPs for multiple samples of initiated with cultures 24, 48 and 72 hours. Regression coefficients estimated from Trendline routine of EXCEL^(C)^.
(TIF)

S3 Fig The effect of the position of the culture in the microtiter plate in estimated MP. Means and standard errors in the estimated MPs for 5 different 10 well columns in the Bioscreen and two plates from the same run, P1 and P2. We found no significant column effect (p~0.5925) in the estimated MP.
(TIFF)

S4 Fig Shaking and wavelength effects. Effect of the shaking routine and wavelength on the estimated MPs (A) and the variance in MP, the ratio of the standard error to the mean for 10 or more wells for each experiment. (B). EC–*E*. *coli*, PA- *Pseudomonas aeruginosa*, PA14, SA- *Staphylococcus aureus* Newman. MIN—minimal medium, LB and MHII are broths. All experiments were performed in the Bioscreen. The control, CON was run the standard way with continuous shaking between reading at 5 minute intervals and a wavelength of 600n. NM540 was run with continuous shaking and a wavelength of 540nm. INT-1, INT-2, and INT-3 were cultures with intermittent shaking; 10 seconds at moderate speed before reading. This experiment was repeated three time to be sure that the unanticipated effect of the shaking routine on the estimated MP for Pseudomonas was repeatable.
(TIF)

S5 Fig Estimation of Lag and Maximum Optical Density. Estimates of the maximum optical density and lag for cultures of different ages, (24, 48 and 72 hours) in broth (LB) and glucose-limited minimal medium (MIN) for **A**. *P*.*aeruginosa*, **B**. *S*. *aureus* in MHII and **C**. *E*. *coli*.
(TIF)

S1 Table Summary of the two-way analyses of variance, run effect and age of culture effect on the estimate MP. Data are presented in Fig 2. This analysis was performed with Graph Pad Prism statistical software. Where F is the ANOVA F-test statistic and P is the probability of having that value of F by chance alone. Highlighted in red are those values for which we obtained statistically significant difference in estimates for MPs between runs, the age of the inoculums and interaction between both parameters.
(TIF)

S2 Table *E*. *coli* Malthusian parameter estimates for different volumes of media in the wells. (A) Growth rates obtained from Bioscreen data using 200, 300, or 350 μl inoculates. Data represent the average of 10 replicas for each culture and media (LB and minimal). The differences in estimated MP for different volumes is statistically significant in both media (p<0.001). (B) Growth rates obtained from BioTek data using 100, 150, 200, or 300 μl as the initial inoculum and the MP is the average of 12 replicas. In both medias, volume differences were significant (p<0.001).
(TIF)

S3 Table Regression analysis of the relationship between the age of the inoculum and MP. (A) For the *S*. *aureus* there is a significant positive correlation with MP and the age of the culture, r = 0.031 with a linear regression coefficient of 2x10^-5^ (9x10^-6^ to 3.3x10^-5^, 95% confidence interval). (B) For the *P*. *aeruginosa* in LB the correlation coefficient is r = -0.25 and the linear regression coefficient -4x10^-5^ (-6 x10^-5^ to 1x10^-5^, 95% confidence interval). The only significant interaction between the age of the inoculum and replica is for *E*. *coli* in minimal medium (p~0.0005) despite no significant age of culture effect (p~0.078).
(TIF)
