# Supplementary material for: Malthusian Parameters as Estimators of the Fitness of Microbes: A Cautionary Tale about the Low Side of High Throughput
Source: PLoS One. 2015 Jun 26;10(6):e0126915. doi: 10.1371/journal.pone.0126915 (PMC4482697; doi:10.1371/journal.pone.0126915)
Supplement: S1 Supporting Information — Method used for the pairwise competition experiments,Calibration experiments not included in the body of this article,The bootstrap method to estimate the confidence intervals in relative maximum exponential growth rates and the Mathematica code for computing these intervals. (DOCX) [file pone.0126915.s001.docx]

**SUPPORTING INFORMATION**

**Malthusian Parameters as Estimators of the Fitness of Microbes: A Cautionary Tale about the Low Side of High Throughput**

Jeniffer Concepción-Acevedo^1^, Howard Weiss^2^, Waqas N. Chaudhry^1,3^ and Bruce R. Levin^1*^

**1- Pairwise competition experiments**

**
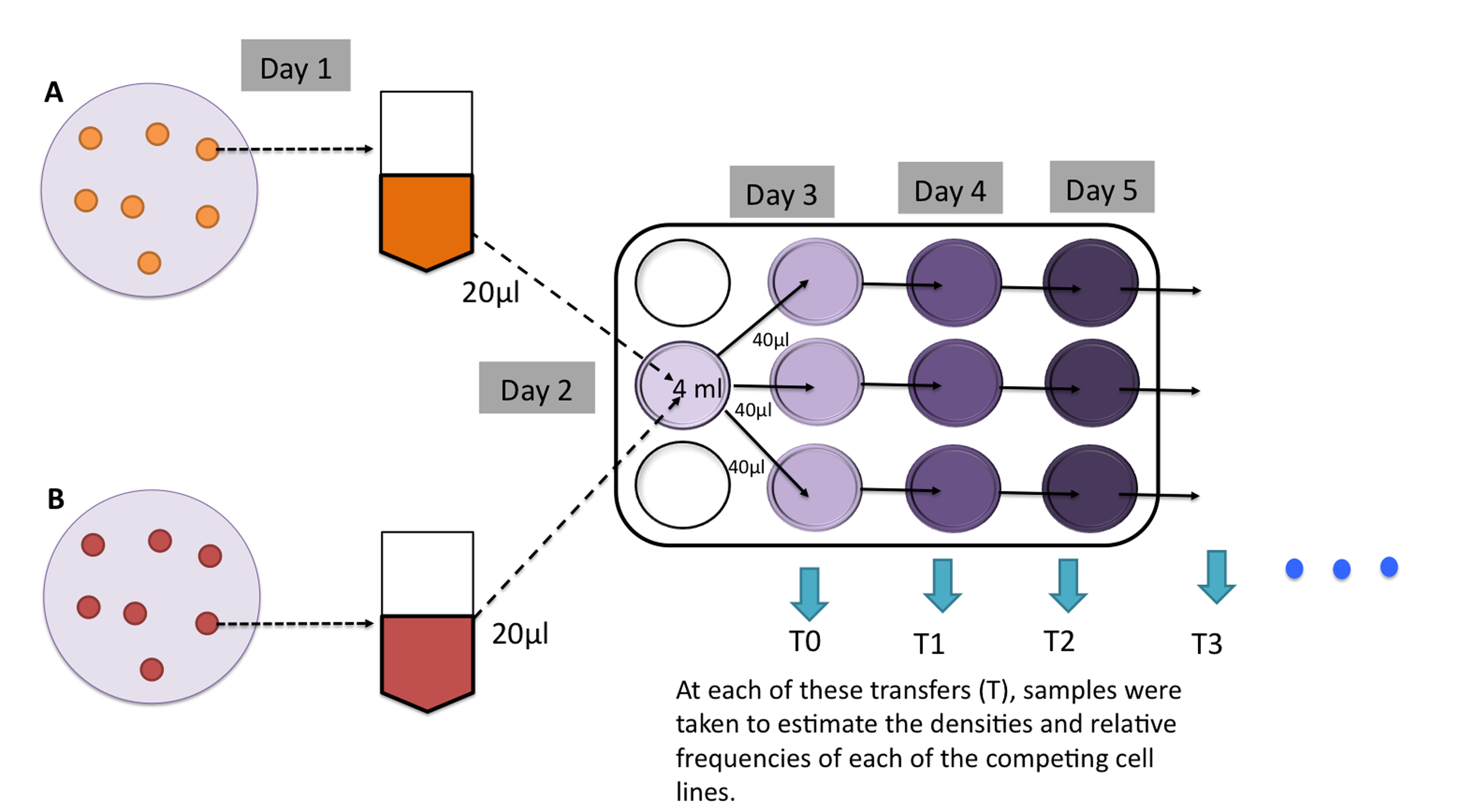
**

**Figure S1.** Diagram of the pairwise competition experiments employed in this study. Overnight stationary phase cultures of the competing strains were mixed on day two, inoculated in media for a ratio 1:100 and grown together for 24 hours. Samples were taken to estimate the densities and relative frequencies of the competing strains, Time 0, and inoculated into 3 wells, 1:100 with media. This process of sampling and dilution in fresh media was repeated for 4 transfers.

**
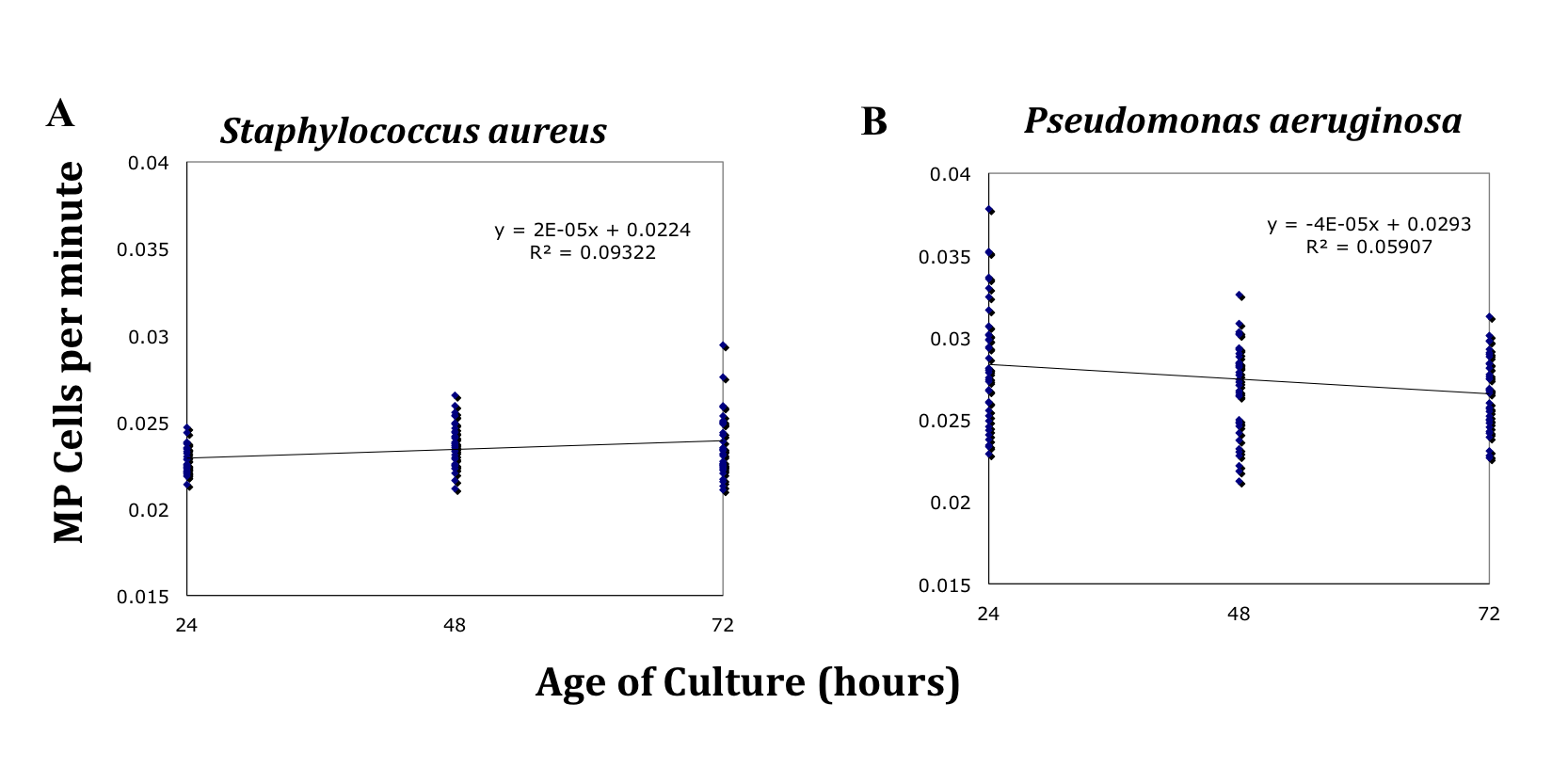
**

**Figure S2.** Effect of the age of the culture on the estimated MPs for *Staphylococcus aureus* (A) and *Pseudomonas aeruginosa* (B). Estimated MPs for multiple samples of initiated with cultures 24, 48 and 72 hours. Regression coefficients estimated from Trendline routine of EXCEL^(C)^.


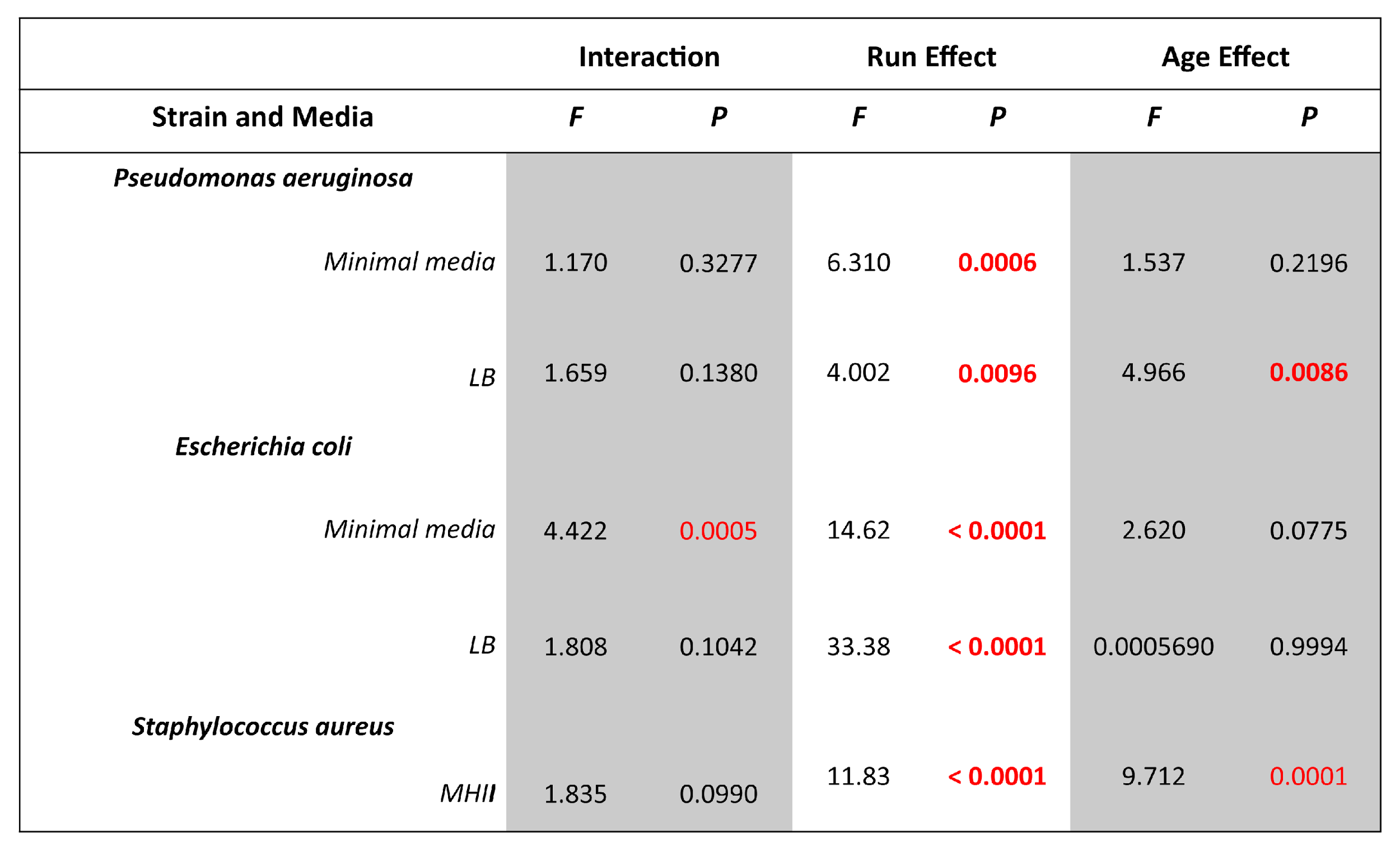


**Table S1.** Summary of the two-way analyses of variance, run effect and age of culture effect on the estimate MP. Data are presented in Figure 2. This analysis was performed with Graph Pad Prism statistical software. Where F is the ANOVA F-test statistic and P is the probability of having that value of F by chance alone. Highlighted in red are those values for which we obtained statistically significant difference in estimates for MPs between runs, the age of the inoculums and interaction between both parameters.

| **Volume (μl)** | **Minimal** | **LB Broth** | | | **ANOVA** | | |
| --- | --- | --- | --- | --- | --- | --- | --- |
|  | **Mean r min^-1^** | **SE** | **Mean r min^-1^** | **SE** |  | **F(2,27)** | **P~** |
| 200 | 0.0188 | 0.0013 | 0.0145 | 0.0003 | **Minimal** | 66.6 | <0.0001 |
| 300 | 0.0155 | 0.0002 | 0.0347 | 0.0003 | **LB** | 820.5 | <0.0001 |
| 350 | 0.0294 | 0.0009 | 0.0341 | 0.0004 |  |  |  |

| **Volume (μl)** | **Minimal** | **LB Broth** | | | **ANOVA** | | |
| --- | --- | --- | --- | --- | --- | --- | --- |
|  | **Mean r min^-1^** | **SE** | **Mean r min^-1^** | **SE** |  | **F(2,27)** | **P~** |
| 100 | 0.013895 | 0.000223 | 0.0261216 | 0.0002436 | **Minimal** | 7.17 | 0.000506 |
| 150 | 0.013510 | 0.000192 | 0.0251375 | 0.0003021 | **LB** | 78.81 | <0.0001 |
| 200 | 0.013458 | 0.000275 | 0.0263208 | 0.0002303 |  |  |  |
| 300 | 0.013835 | 0.000273 | 0.0308541 | 0.0003554 |  |  |  |

**Table S2.** *E. coli* Malthusian parameter estimates for different volumes of media in the wells. Top: Growth rates obtained from Bioscreen^TM^ data using 200, 300, or 350 μl inoculates. Data represent the average of 10 replicas for each culture and media (LB and minimal). The differences in estimated MP for different volumes is statistically significant in both media (p<0.001). Bottom: Growth rates obtained from BioTek^TM^ data using 100, 150, 200, or 300 μl as the initial inoculum and the MP is the average of 12 replicas. In both medias, volume differences were significant (p<0.001).

.

**C.**


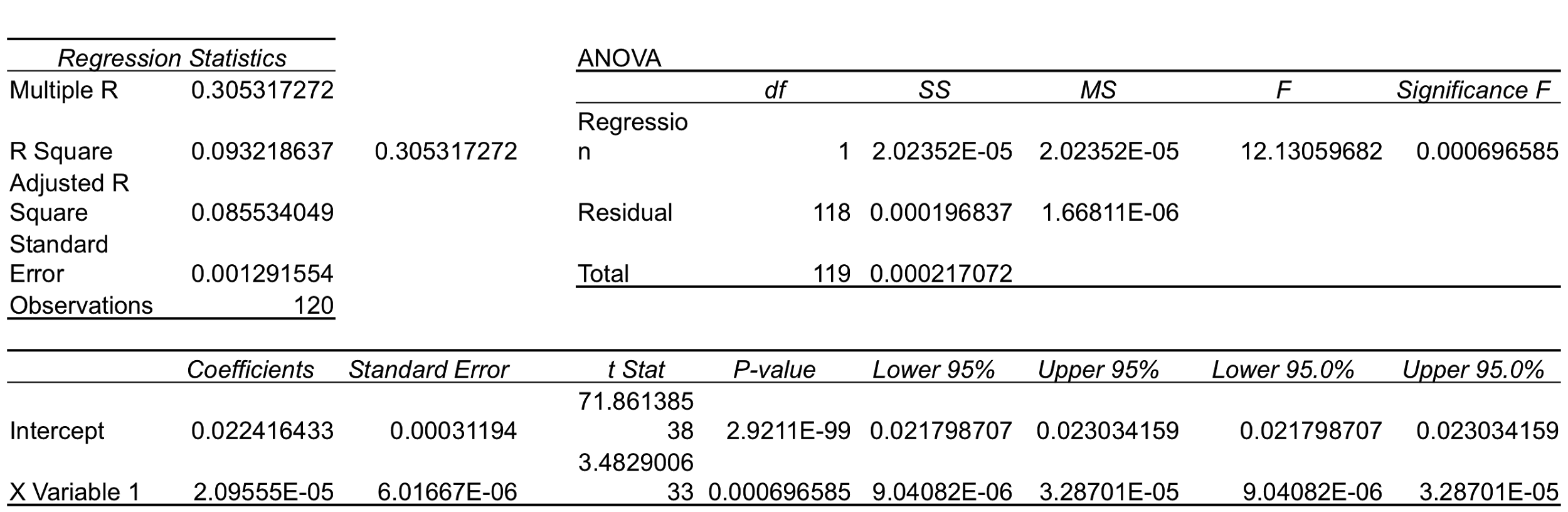


**D.**


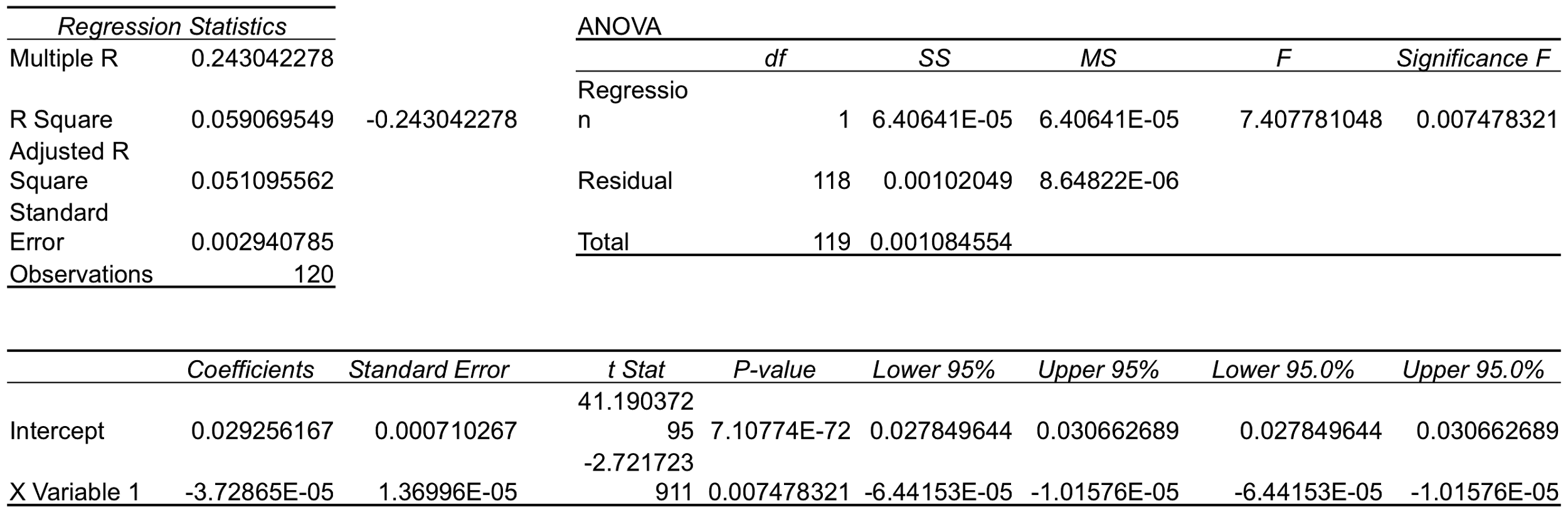


**Table S3.** Regression analysis of the relationship between the age of the inoculum and MP. For the *S. aureus* there is a significant positive correlation with MP and the age of the culture, r=0.031 with a linear regression coefficient of 2x10^-5^ (9x10^-6^ to 3.3x10^-5^, 95% confidence interval). For the *P. aeruginosa* in LB the correlation coefficient is r=-0.25 and the linear regression coefficient -4x10^-5^ (-6 x10^-5^ to 1x10^-5^, 95% confidence interval). The only significant interaction between the age of the inoculum and replica is for E. coli in minimal medium (p~0.0005) despite no significant age of culture effect (p~0.078).

**3**


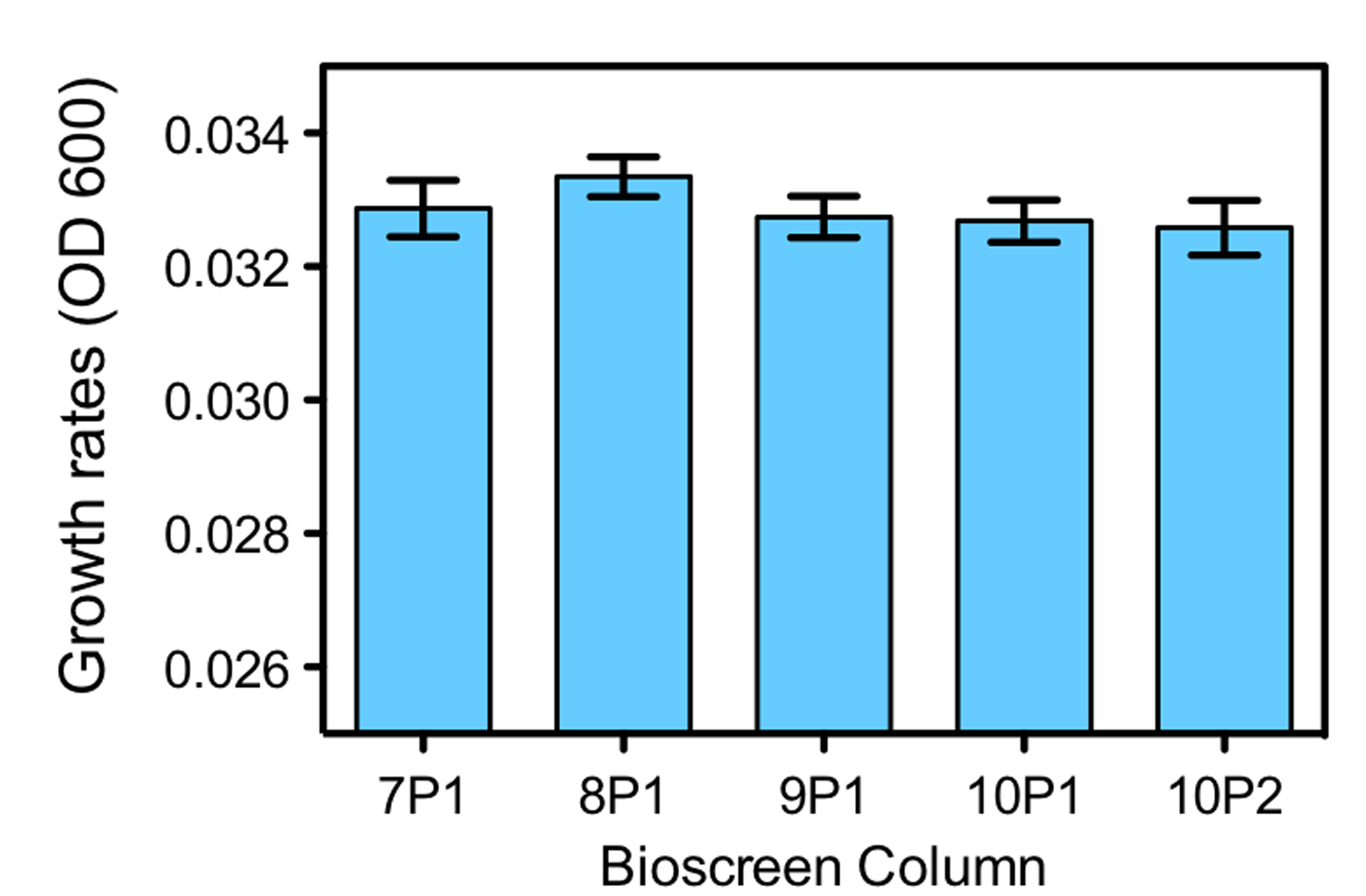


**Figure S3.** Position effects: Means and standard errors in the estimated MPs for 5 different 10 well columns in the Bioscreen^TM^ and two plates from the same run, P1 and P2. We found no significant column effect (p~0.5925) in the estimated MP.


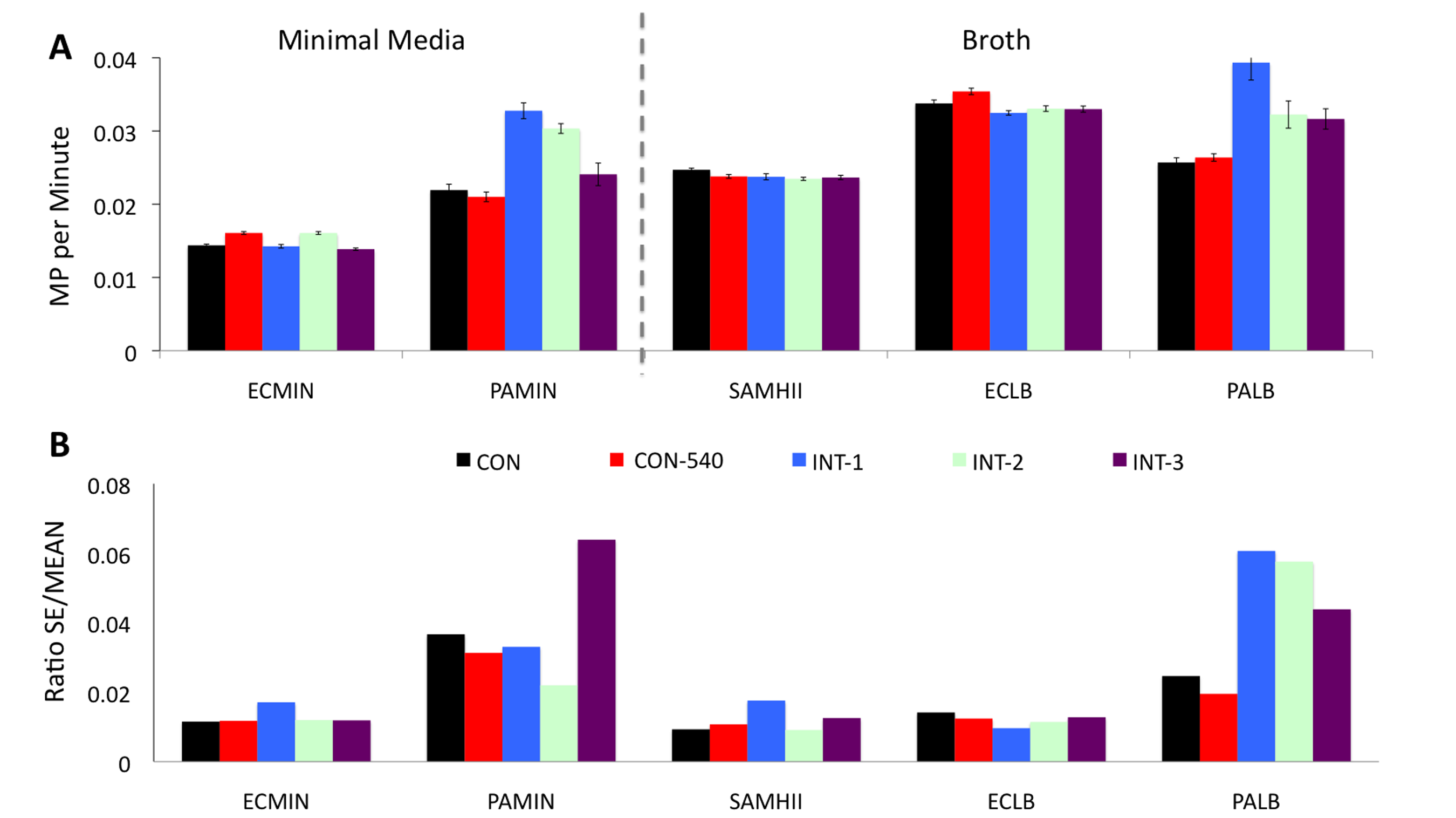


**Figure S4**. Effect of the shaking routine and wavelength on the estimated MPs (A) and the variance in MP, the ratio of the standard error to the mean for 10 or more wells for each experiment. (B). EC – *E. coli*, PA- *Pseudomonas aeruginosa*, PA14, SA- *Staphylococcus aureus* Newman. MIN – minimal medium, LB and MHII are broths. All experiments were performed in the Bioscreen^TM^. The control, CON was run the standard way with continuous shaking between reading at 5minute intervals and a wavelength of 600n. NM540 was run with continuous shaking and a wavelength of 540nm. INT-1, INT-2, and INT-3 were cultures with intermittent shaking; 10 seconds at moderate speed before reading. This experiment was repeated three times to be sure that the unanticipated effect of the shaking routine on the estimated MP for Pseudomonas was repeatable.

**5- Bootstrap estimation of the confidence intervals for the relative (with respect to wild type) maximum exponential growth rates.**

Let WT denote the observed values of the growth rates of the wild type strain and let M denote the observed values of the growth rates of the Mutant strain. Although not essential, assume the same number of observations for both, say n. Consider all possible ordered n^2^ pairs of the two strains (“M’’, “WT”) and for each pair compute their ratio. Take 1000 random (bootstrap) samples of size n from these ratios, and for each sample compute the mean. The 99% confidence interval is defined as the (.005, .995) quartile of these 1000 values.

For identical samples, we compute the 99% confidence intervals as described above for the BioTek and BioScreen. If they are disjoint, we say that there is a significant difference between the two machines at the 99% level of confidence.

**Mathematica^TM^ code to compute the above described CIs**

Num = 12;
WT = {0.02122, 0.02095, 0.02157, 0.02074, 0.02055, 0.02231, 0.02057,
 0.02114, 0.02178, 0.01831, 0.02254, 0.02116};
M = {0.02186, 0.02256, 0.02088, 0.02335, 0.02535, 0.02531, 0.02467,
 0.02346, 0.02278, 0.02586, 0.02962, 0.02495};
A = Tuples[{M, WT}];
B = Table[A[[j]][[1]]/A[[j]][[2]], {j, 1, Num^2}];
T = Table[Mean[RandomChoice[B, Num]], {j, 1, 1000}];
Needs["HypothesisTesting`"]
Quantile[T, {.005, .995}]

**6- Estimation of Lag and Maximum Optical Density.**


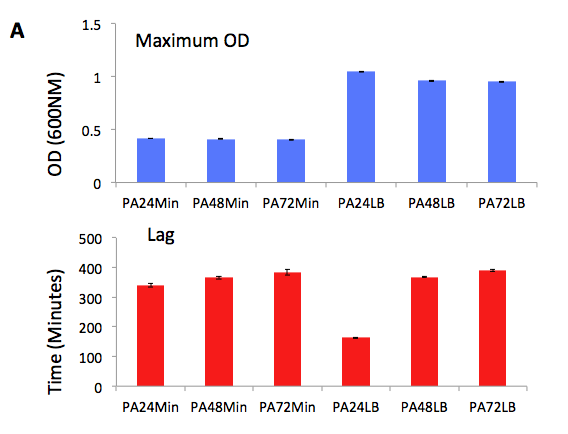


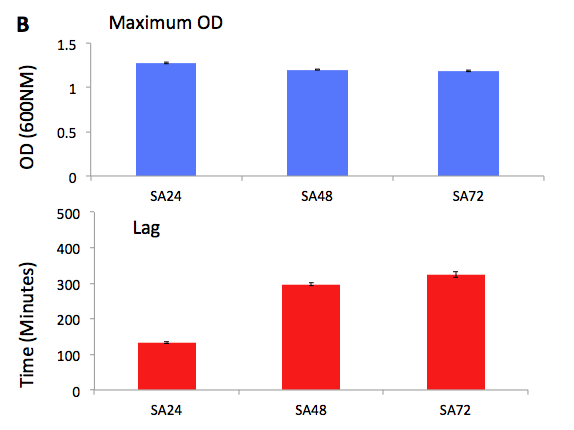


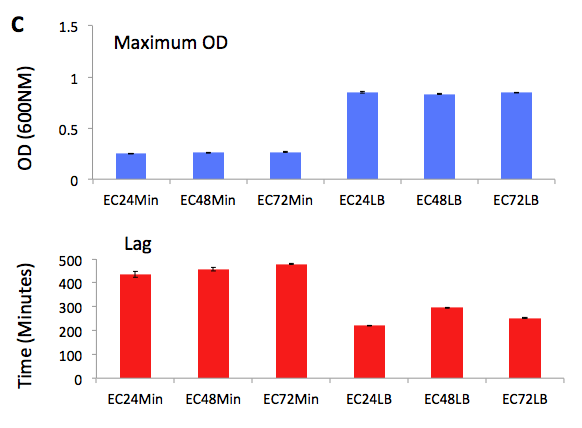


**Supplemental Figure 5.** Estimates of the maximum optical density and lag for cultures of different ages, (24, 48 and 72 hours) in broth (LB) and glucose-limited minimal medium (MIN) for **A***. P.aeruginosa,* **B.** *S. aureus* in MHII and **C**. *E. coli.*
